# Enhanced Angiogenesis by 11βHSD1 Blockage Is Insufficient to Improve Reperfusion Following Hindlimb Ischaemia

**DOI:** 10.3389/fcvm.2021.795823

**Published:** 2022-01-12

**Authors:** Junxi Wu, Eileen Miller, Callam Davidson, Brian R. Walker, Patrick W. F. Hadoke

**Affiliations:** ^1^The Queen's Medical Research Institute, University/BHF Centre for Cardiovascular Science, University of Edinburgh, Edinburgh, United Kingdom; ^2^Department of Biomedical Engineering, University of Strathclyde, Glasgow, United Kingdom

**Keywords:** 11βHSD1, critical hindlimb ischaemia, angiogenesis, reperfusion, arteriogenesis

## Abstract

**Background:** Critical limb ischaemia (CLI), which is estimated to affect 2 million people in the United States, reduces quality of life, is associated with high morbidity and mortality, and has limited treatment options. Direct stimulation of angiogenesis using proangiogenic growth factors has been investigated as a therapeutic strategy to improve reperfusion in the ischaemic leg. Despite positive outcomes in animal studies, there has been little success in clinical translation. This investigation addressed the hypothesis that angiogenesis could be stimulated indirectly in the ischaemic hindlimb by blocking 11β-hydroxysteroid dehydrogenase 1 (11βHSD1)-mediated reactivation of anti-angiogenic glucocorticoids.

**Method and Results:** Corticosterone suppressed *ex vivo* angiogenesis in the mouse aortic ring assay. 11βHSD1 deletion (Hsd11b1^Del1/Del1^) or pharmacological inhibition (with 300 nM UE2316) which block the reactivation of glucocorticoid (i.e., the conversion of 11-dehydrocorticosterone (11DHC) to bioactive corticosterone) significantly reduced 11DHC-induced suppression of angiogenesis. In a sponge implantation model, 11βHSD1 deletion, but not pharmacological inhibition, enhanced inflammation-induced angiogenesis. By contrast, in the mouse hindlimb ischaemia model, post-ischaemic reperfusion and vascular density were not affected by either deletion or pharmacological inhibition of 11βHSD1 in young or aged mice. 3D vascular imaging suggested that hind limb reperfusion in the 1st week following induction of ischaemia may be driven by the rapid expansion of collateral arteries rather than by angiogenesis.

**Conclusion:** 11βHSD1-mediated glucocorticoid reactivation suppressed angiogenesis *ex vivo* and *in vivo*. However, regulation of angiogenesis alone was insufficient to promote reperfusion in hindlimb ischaemia. Future investigation of post-ischaemic reperfusion should include other aspects of systemic vascular remodeling including arteriogenesis and collateral formation.

## Introduction

Critical limb ischaemia (CLI), caused by blockage of conduit arteries supplying the lower extremities, has been estimated to affect ~2 million people in the United States (1). It produces significant clinical symptoms including claudication, rest pain and tissue gangrene and has a poor prognosis with reduced quality of life and high morbidity and mortality (1 year mortality ranging from 15 to 40%) (1). Treatment of CLI relies heavily on revascularisation techniques (bypass surgery and endovascular stenting) with the only medical interventions available targeting co-existing cardiovascular risk factors (1). Despite these options, amputation is common in these patients (>15–20% at 1 year) and is a significant risk factor for in-hospital mortality (1, 2). Insufficient post-ischaemic vascular regeneration and remodeling, particularly in elderly patients, contributes to poor reperfusion and increased risk of amputation (2–4). It is well-known that hypoxia-induced angiogenesis is largely driven by activation of HIF-1α signaling pathway that increases the expression of pro-angiogenic factors including vascular endothelial growth factor (VEGF) (2, 5). Hypoxia-induced angiogenesis is insufficient to improve reperfusion the ischaemic leg in many CLI patients. Therefore, research has been focused on development medical interventions of direct stimulation of angiogenesis (e.g., VEGF-based gene therapy) as a non-surgical therapy for “no-option” CLI patients. However, little success has been achieved in clinical translation (2, 6, 7). Alternative therapeutic strategies are urgently needed for CLI.

Glucocorticoids are well-documented for their ability to supress angiogenesis (8–11). Excessive circulating glucocorticoids (as seen in Cushing's disease or long-term glucocorticoid therapy) can lead to femoral head necrosis due to suppression of angiogenesis (12, 13). There is evidence suggesting that hypoxia potentiates glucocorticoid signaling *via* upregulation of glucocorticoid receptor (GR) expression and sensitivity (4) which could exacerbate the anti-angiogenic effect of glucocorticoid in CLI. Inhibition of glucocorticoid signaling, therefore, may provide an alternative therapeutic target to upregulate angiogenesis in CLI. Glucocorticoids are regulated at both systemic and local levels in the body. Systemic glucocorticoid inhibition is known to cause severe side effects; over 20% of the patients receiving mifepristone (a glucocorticoid receptor (GR) antagonist) reported adverse reactions associated with disruption of hypothalamic-pituitary-adrenal axis homeostasis (14). By contrast, tissue-specific regulation of glucocorticoid metabolism by 11β-hydroxysteroid dehydrogenases (11βHSD1 and 11βHSD2) has minimal impact on systemic level of glucocorticoids (15–17). Bioactive glucocorticoids are inactivated mainly by renal 11βHSD2 (which converts cortisol to cortisone in humans and corticosterone to 11-dehydrocorticosterone (11DHC) in rodents), whereas 11βHSD1 reactivates glucocorticoids in glucocorticoid target tissues (such as muscle) to enhance their local action. Inhibition of 11βHSD1, therefore, provides a potential strategy to reduce local glucocorticoid regeneration and enhance angiogenesis. In a mouse model of diet-induced obesity, 11βHSD1 deficiency reduced inflammation and increased angiogenesis in adipose tissue (18, 19). Similar pro-angiogenic effects were also observed in a murine myocardial infarction model following genetic deletion or pharmacological inhibition of 11βHSD1 (20, 21). Notably, genetic ablation of 11βHSD1 in the myocardial infarction model did not alter the circulating glucocorticoid concentration throughout the course of the study, but acted locally in the myocardium to increase macrophage recruitment, pro-angiogenic IL-8, vessel density, and cardiac function (20). In addition, there is evidence showing that pro-inflammatory cytokine TNF-α potently upregulated 11βHSD1 expression and enhanced local glucocorticoid reactivation in both human and rodent skeletal muscle, whereas genetic ablation of 11βHSD1 completely abolished glucocorticoid regeneration in muscle (22, 23). These evidence suggest that inhibition of 11βHSD1 may serve as a potential therapeutic strategy to reduce intra-muscular glucocorticoids and increase angiogenesis in the ischaemic muscle in CLI patients. Therefore, the current investigation addressed the hypothesis that genetic ablation or pharmacological inhibition of 11βHSD1 would enhance angiogenesis and improve reperfusion in a murine model of hindlimb ischaemia.

## Methods

### Animal Study

Animal experiments were performed in accordance both with Directive 2010/63/EU of the European Parliament and with the UK Home Office Animal (Scientific Procedures) Act 1986, and licensed under Project License PPL 60/4523. All procedures were approved by the University of Edinburgh ethical review committee (Animal Welfare and Ethical review body). 11βHSD1-deficient mice (Hsd11b1^Del1/Del1^) were previously generated on a C57BL/6 background (24) and maintained in the University of Edinburgh. Wild type C57BL/6 mice were purchased from Charles River, UK. Mice were housed in groups (2–5 per cage) under controlled conditions: 12 h light/darkness cycle at 21°C with free access to standard rodent chow and water unless otherwise specified. Adult male mice (> 10 weeks old) were used to investigate *in vivo* vascular remodeling (25, 26). The age of mice is specified in each experiment.

### Aortic Ring Assay

The aortic ring assay was employed to study *ex vivo* angiogenesis, as previously described (27). Briefly, mice (8–10 weeks old) were culled by asphyxiation in CO_2_ and the descending thoracic aorta isolated. The aortae were cut into 18 rings and each ring embedded in 100 μl of collagen gel [1% collagen in Opti-MEM culture medium (Gibco, UK. Cat. No. 51985–026)] in a 96-well plate. 200 μl of Opti-MEM containing penicillin-streptomycin, 2% fetal bovine serum (FBS) and 5 ng/ml vascular endothelial growth factor (VEGF. PEPROTECH, UK. Cat. No.450-32) were added on top of the gel. On day 3 and 5, culture media were changed using Opti-MEM plus 5 ng/ml VEGF with no FBS. The number of tube-like structures in each well was manually counted on day 7.

Pharmacological inhibition of 11βHSD1 was achieved by using a new generation of 11βHSD1 inhibitor UE2316 ([4-(2-chlorophenyl)-4-fluoro-1-piperidinyl][5-(1H-pyrazol-4-yl)-3-thienyl]-methanone, synthesized by High Force Ltd., UK) which has higher potency than the old generation inhibitor UE1961 (28–31). UE2316 was dissolved in DMSO and diluted to a concentration of 300 nM using culture medium. The final concentration of DMSO was <0.06% vol/vol. Corticosterone and 11DHC were dissolved in ethanol and diluted to a concentration of 300 nM using culture medium, respectively (8). The final concentration of ethanol was <0.3% vol/vol. Experiments were performed in triplicate. UE2316, Corticosterone and 11DHC were incubated with aortic rings for 7 days.

### Sponge Implant Model

The effect of pharmacological inhibition and genetic ablation of 11βHSD1 on *in vivo* angiogenesis was investigated using a sponge implant model (8). Mice (10–12 weeks old) were anesthetized with isoflurane and two sterilized sponge blocks (1.5 × 1 × 1 cm; Caligen Foam, Accrington, Lancashire, UK) inserted subcutaneously in each flank (8). Twenty-one days after implantation, mice were euthanised with CO_2_ and sponges removed. One sponge was fixed in 10% formalin for histology study. Vascular density was quantified using a Chalkley score (32) and by quantifying vessels staining positive for anti-smooth muscle alpha-actin (SMA^+^). The other sponge was frozen at −80°C for RNA extraction.

Pharmacological inhibition of 11βHSD1 was achieved by using a UE2316-blended chow diet (0.0175% w/w) from 7 days before surgery until the end of the experiment, with an estimated dose of 25–30 mg/kg/day (29). The effects of genetic ablation of 11βHSD1 were studied using Hsd11b1^Del1/Del1^ mice (24). Hsd11b1^Del1/Del1^ and wild type control C57BL/6 mice were fed on normal chow diet. Detailed experiment design is illustrated in Supplementary Figure 1A.

### Hindlimb Ischaemia Model and Laser Doppler Imaging

A murine model of unilateral femoral artery ligation (26, 33) was employed to investigate the role of 11βHSD1 in vascular remodeling and reperfusion following acute hindlimb ischaemia. Briefly, mice were anesthetised by inhalation of isoflurane (5% for induction and 2–3% for maintenance) with appropriate analgesic cover (buprenorphine; 0.05 mg/kg body weight, s.c.). Depth of anesthesia was indicated by loss of the pedal withdrawal reflex. The femoral artery in the left leg was ligated distal to the inguinal ligament and the saphenous artery ligated distal to the saphenous/popliteal bifurcation. The femoral artery was cauterized distal to the femoral ligature. Wounds were closed using a 6–0 Mersilk suture and mice were allowed to recover for 28 days. Blood flow in the foot pad was measured with a Moor Infrared Laser Doppler Imager (Moor Instruments, UK) immediately before and after femoral artery ligation and at 3, 7, 14, 21, and 28 days after surgery. To control the variation of systemic perfusion caused by handling stress and fluctuation of body temperature in individual mouse, blood perfusion in the ischaemic foot pad (the mean signal intensity of the selected area) was normalized to perfusion in the tail, as described before (26). The choice of normalization to tail, rather than the non-ischaemic leg, was because femoral artery ligation induces irregular fluctuation in perfusion in the non-ischaemic leg in the first few days (data not shown). Mice were killed by asphyxiation in CO_2_ on post-ischaemic day 28. Major organs including liver, spleen, heart, thymus, kidneys, and adrenal glands were collected and weighed. Gastrocnemius muscle was harvested both from ischaemic and from control legs. Each muscle was cut into two pieces. One half was frozen at −80°C and the other half fixed in 10% neutral buffered formalin for 24 h. The effects of 11βHSD1 inhibition with UE2316 and genetic ablation were studied both in young adults (male, 13 ± 0.1 weeks old) and in aged mice (male, 33.5 ± 0.5 weeks). Detailed experimental design is illustrated in Supplementary Figures 1B,C. In both young and aged mice, no differences were observed in food intake, body weight or internal organ weight across all groups. The dose of UE2316, calculated from average daily food intake, was 27 mg/Kg/day in both young and aged mice (Supplementary Figure 2).

### Histology and Immunostaining

Cross-sections from the middle of the gastrocnemius muscle or the sponge implant were used for haematoxylin and eosin staining. Total blood vessel number [Isolectin B4 (IB4), Life Technologies] and number of mature arteries/veins (SMA antibody, Sigma-Aldrich) were identified by immunostaining. To quantify the vessel density, a whole cross-section of gastrocnemius muscle was tile-scanned at x200 magnification (Slide scanner Axio Scan.Z1, Zeiss). The densities of IB4^+^ and SMA^+^ vessels were quantified using ImageJ software. For sponge implants, cross-sections were tile-scanned and SMA^+^ vessel density quantified. In addition, the vessel density in sponge implants was determined by Chalkley count, as described previously (8).

### Quantitative PCR

RNA was isolated by homogenisation in QIAzol Lysis Reagent (Qiagen), followed by extraction with chloroform (Sigma-Aldrich) and purification using a RNeasy Mini Kit (Qiagen). RNA concentration and quality were measured using a NanoDrop 1000 Spectrophotometer (Thermo Scientific). RNA samples were reverse transcribed to cDNA using a Quantitect Reverse Transcription Kit (Qiagen). Quantitative PCR was performed on the Light Cycler 480 system (Roche Diagnostics) using TaqMan Gene Expression assays (Life Technologies). A standard curve for each primer-probe set was generated by serial dilution of pooled cDNA from several animals. Each sample was run in triplicate, and the mean values of the triplicates were used to calculate transcript level from the standard curve of the same tissue. The expression level of tested genes was normalized to housekeeping genes *Actb* and *Gapdh*.

Imaging and quantification of arteriogenesis (collateral growth) in ischaemic hindlimb C57BL/6 mice (male, 10–20 weeks old) were randomly assigned to 5 groups (*n* = 3 for each group) and subjected to unilateral femoral artery ligation as described above. Blood perfusion in the ischaemic foot (by laser Doppler, as described above) was measured in each group at separate time points at pre-surgery, post-surgery, day 1, day3, and day 7 (*n* = 3 for each time point). The mice were killed by asphyxiation in CO_2_, immediately after laser Doppler imaging. The abdominal vena cava was cut open for exsanguination. 20–40 ml of Phosphate buffered saline (PBS) were injected into the left ventricle of the heart to flush out the blood in the circulation. Low viscosity resin (Biodur® E20 Plus) was then injected into the left ventricle to cast the vascular tree. This resin is too viscous to pass the capillaries and, therefore, only casts the arterial tree. The ischaemic leg was then harvested and processed for optical projection tomography (OPT), as described (34), to illustrate the whole arterial tree in the hindlimb. New collateral arteries were identified by comparing pre-surgical and post-surgical images. The diameter of the biggest collateral artery from each leg was measured using ImageJ.

### Statistics

Data were expressed as mean ± standard error of the mean (SEM), where n refers to the number of mice. Data between two groups were analyzed using unpaired Student's *t*-test. Data from multiple groups were analyzed using one-way or two-way ANOVA with Bonferroni's *post-hoc* test, as appropriate. Statistical analyses were performed using GraphPad Prism v6.04. Differences were considered significant when *p* < 0.05.

## Results

### Blocking 11βHSD1 Activity Restored *ex vivo* Angiogenesis in the Presence of 11-DHC

**Figure 1:**
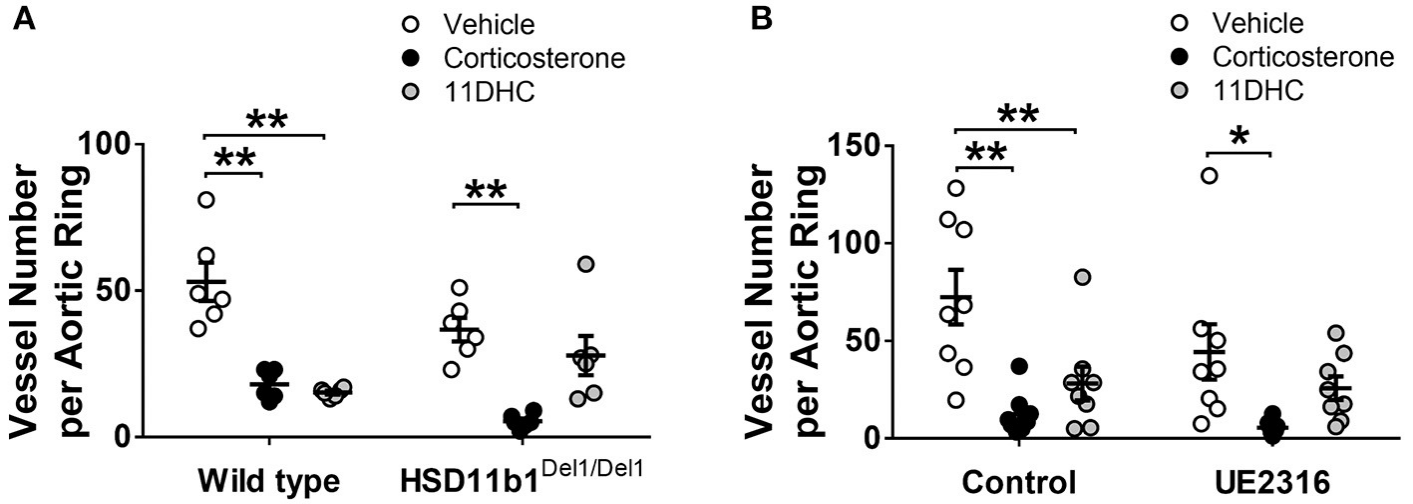
Genetic ablation and pharmacological inhibition of 11βHSD1 blocked glucocorticoid reactivation and increased angiogenesis in an aortic ring assay. **(A)** The bioactive corticosterone (300 nM for 7 days) inhibited angiogenesis in all groups. The Inert 11DHC (300 nM for 7 days) inhibited angiogenesis only in the wild type group, but not in HSD11b1^Del1/Del1^ (*n* = 6). **(B)** In wild type aortic rings, Inhibition of 11βHSD1 (UE2316, 300 nM for 7 days) abolished the anti-angiogenic effect of 11DHC (*n* = 8). Bars indicate mean ± SEM. **P < 0.01, *P < 0.05, by two-way ANOVA plus Bonferroni's *post hoc* test.

In the absence of glucocorticoids, FBS and VEGF-stimulated angiogenesis (in vehicle groups) in the aortic ring assay was not affected by genetic ablation or pharmacological inhibition of 11βHSD1. The angiogenesis was significantly suppressed by corticosterone treatment (300 nM) (Figure 1). When exposed to the inert 11DHC (300 nM), angiogenesis was suppressed in aortic rings from wild type but not in those from Hsd11b1^Del1/Del1^ mice (Figure 1A). Similar to genetic ablation, incubation of aortic rings with the 11βHSD1 antagonist UE2316 (300 nM) somewhat prevented 11DHC-mediated inhibition of angiogenesis in wild type aortic rings (Figure 1B). These data confirmed the critical role of 11βHSD1 in reactivation of local glucocorticoids which directly suppress angiogenesis, whereas blockage of 11βHSD1 pathway restored *ex vivo* angiogenesis.

### 11βHSD1 Deficiency, but Not Pharmacological Inhibition, Increased *in vivo* Angiogenesis in a Sponge-Implant Model

*In vivo* angiogenesis occurs in a more complex environment than angiogenesis in the *ex vivo* aortic ring model and is often associated with inflammation. The sponge implantation model was used to investigate the effect of 11βHSD1 on *in vivo* angiogenesis. Twenty-one days following surgery, all sponge implants demonstrated some extent of vascularization. The anti-CD31 antibody and isolectin B4 were first attempted to visualize the blood vessels. However, the inflammatory cells in the sponge generated very strong non-specific staining that obscured the vessel count (data not shown). We therefore adopted the Chalkley count and immunostaining against SMA to quantify vessel number in sponge blocks.

**Figure 2:**
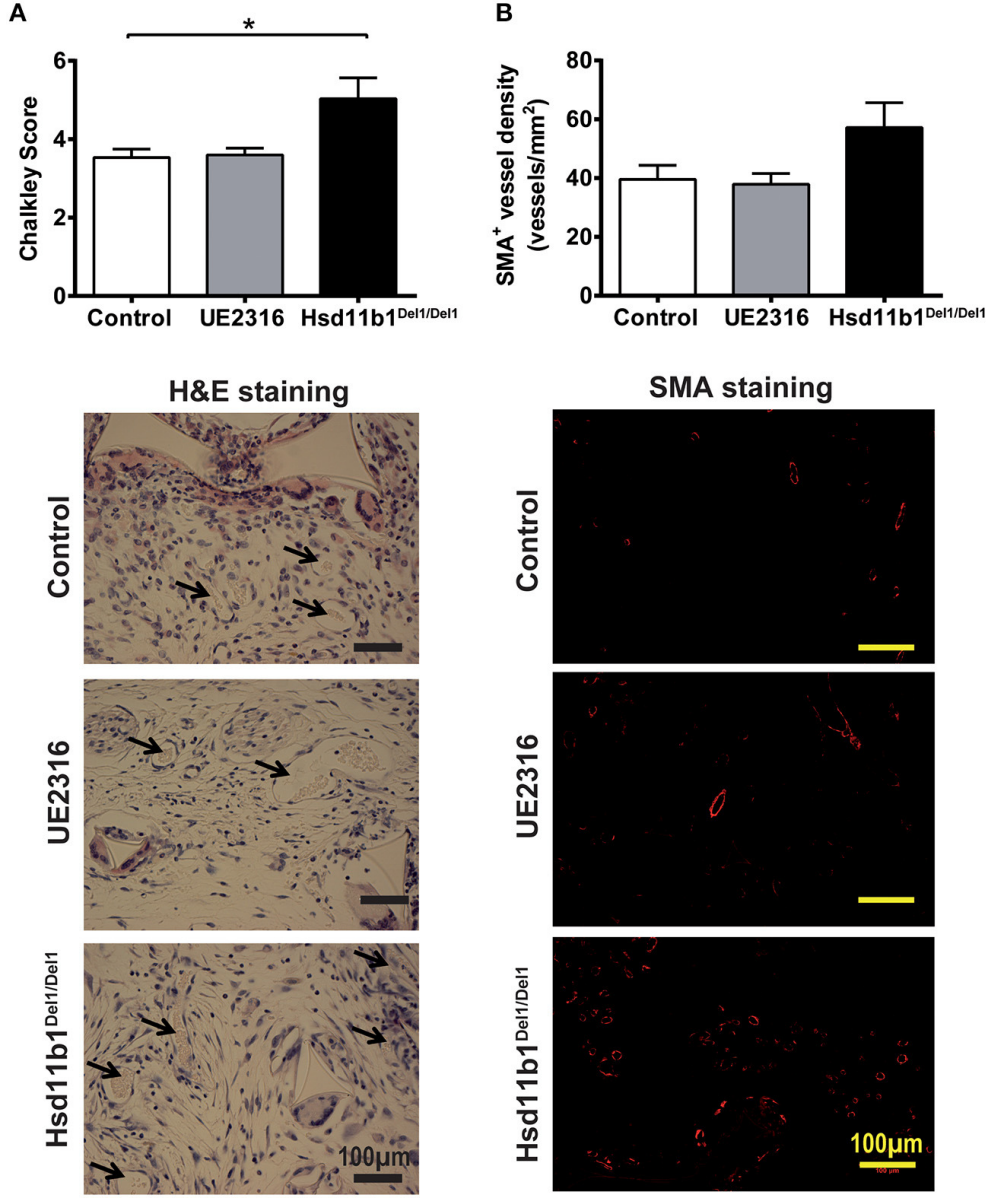
11βHSD1 deficiency, but not UE2316 treatment, promoted *in vivo* angiogenesis in a sponge implant model. **(A)** New vessel formation in subcutaneously implanted sponge blocks were identified based on vascular morphology and quantified using Chalkley count. 11βHSD1 deficiency, but not UE2316 treatment (27 mg/kg/day), had significantly higher Chalkley score vs. wild type control. **(B)** Arteries in the sponge block was visualized using immunostaining against SMA+. The levels of artery density showed a similar pattern to total vessel count (by Chalkley score), but no statistical significance was reached. *P < 0.05, by one-way ANOVA plus Bonferroni's *post-hoc* test (*n* = 10).

Chalkley counts in sponges from Hsd11b1^Del1/Del1^ mice were significantly higher than those in control and UE2316-treated groups (Control 3.53 ± 0.21 vs. UE2316 3.60 ± 0.17 vs. Hsd11b1^Del1/Del1^ 5.03 ± 0.54; *p* < 0.05 by One-Way ANOVA plus Bonferroni's *post-hoc* test) (Figure 2A). A similar pattern was observed in SMA^+^ vessel density but this was not statistically significant (Figure 2B). mRNA expression of ischaemia and angiogenesis-related growth factors (*Hif1a, Pdgfrb, Vegfa, and Fgf2*), endothelial cell markers (*Pecam1, Vwf, and Cdh5*), smooth muscle cell markers (*Myh11, Cnn1, and Acta2*) and inflammatory factors (*Ccl2, Il1b, Il6, and Tnf* ) did not differ between groups (Supplementary Figure 3A).

### Targeting 11βHSD1 Activity Did Not Increase Reperfusion in Ischaemic Hindlimb in Young Adult Mice

**Figure 3:**
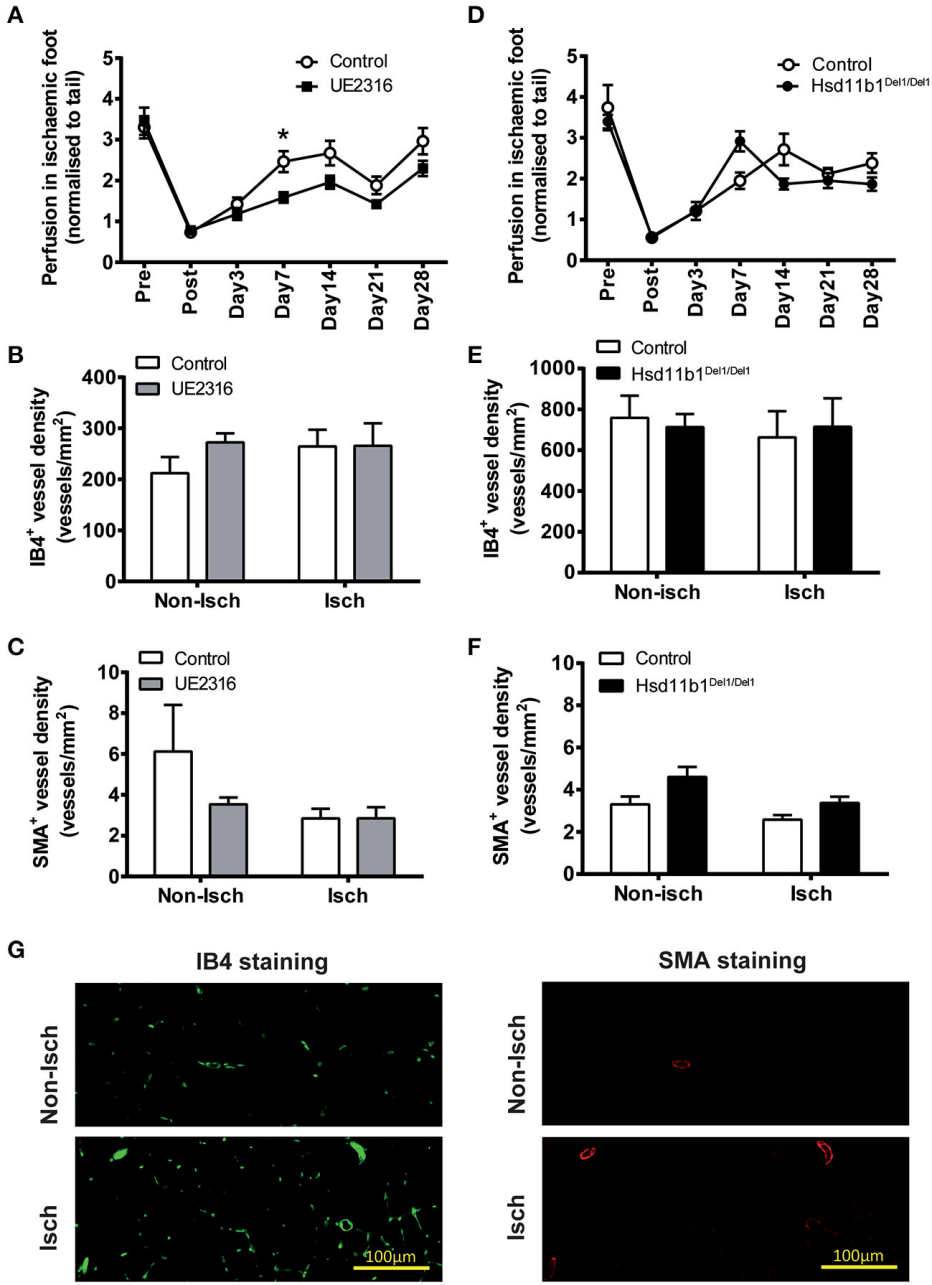
UE2316 treatment and 11βHSD1 deficiency had limited effect on hindlimb ischaemia in young adult mice. **(A)** Unilateral femoral artery ligation produced a dramatic reduction in hindlimb perfusion, followed by a quick reperfusion peaking at 7–14 days. UE2316 treatment (27 mg/kg/day) was associated with a transient reduction in reperfusion only at day 7. At day 28, none of hindlimb perfusion **(A)**, capillary density **(B)** and artery density **(C)** in gastrocnemius muscles were affected by UE2316 treatment. **(D–F)** 11βHSD1 deficiency had no impact on post-ischaemic reperfusion, capillary or artery density in gastrocnemius muscles. **(G)** Representative images of capillaries (green color) and arteries (red color) in gastrocnemius muscles. Symbols are mean ± SEM; columns are mean + SEM. *P < 0.05, by two-way ANOVA plus Bonferroni's *post-hoc* test (*n* = 10).

Femoral artery ligation induced an 80% reduction in foot-pad perfusion in young adult mice (male, 13.0 ± 0.1 weeks old) (Figures 3A,D). Reperfusion increased in all groups over a period of 7–14 days and stabilized afterwards. In mice receiving UE2316 (27 mg/kg/day) treatment reperfusion was transiently blunted at day 7 (Control 2.46 ± 0.24 vs. UE23161.58 ± 0.12; *p* < 0.05 by two-way ANOVA with Bonferroni's *post-hoc* test) but this difference was no longer significant by day 14 (Figure 3A). Vascular density in the gastrocnemius muscle, whether measured as density of IB4 positive (Figure 3B) or smooth muscle actin positive vessels (Figure 3C), was unchanged in the UE2316-treated group at 28 days. 11βHSD1 deficiency (Hsd11b1^Del1/Del1^) had no effect on post-ischaemic reperfusion or vascular density (Figures 3D–F). At transcription level, the expression profile of ischaemia marker (*Hif1a*), angiogenesis-related growth factors (*Pdgfrb, Vegfa, and Fgf2*), endothelial cell markers (*Pecam1, Vwf, and Cdh5*), smooth muscle cell markers (*Myh11, Cnn1, and Acta2*) and inflammatory factors (*Ccl2, Il1b, Il6, and Tnf*) were similar between Hsd11b1^Del1/Del1^ and control. Expression levels of inflammatory factors were negligible in all groups (Supplementary Figure 3B, upper panel). Interestingly, the expression levels of *Vegfa* and *Cnn1* in the control group were statistically higher than those in the UE2316-treated group (Supplementary Figure 3B, lower panel). However, it was not clear whether this was a result of UE2316 treatment, or an artifact due to the suspiciously high levels of *Vegfa* and *Cnn1* in the control group.

### Targeting 11βHSD1 Activity Did Not Increase Reperfusion in Ischaemic Hindlimb in Aged Mice

**Figure 4:**
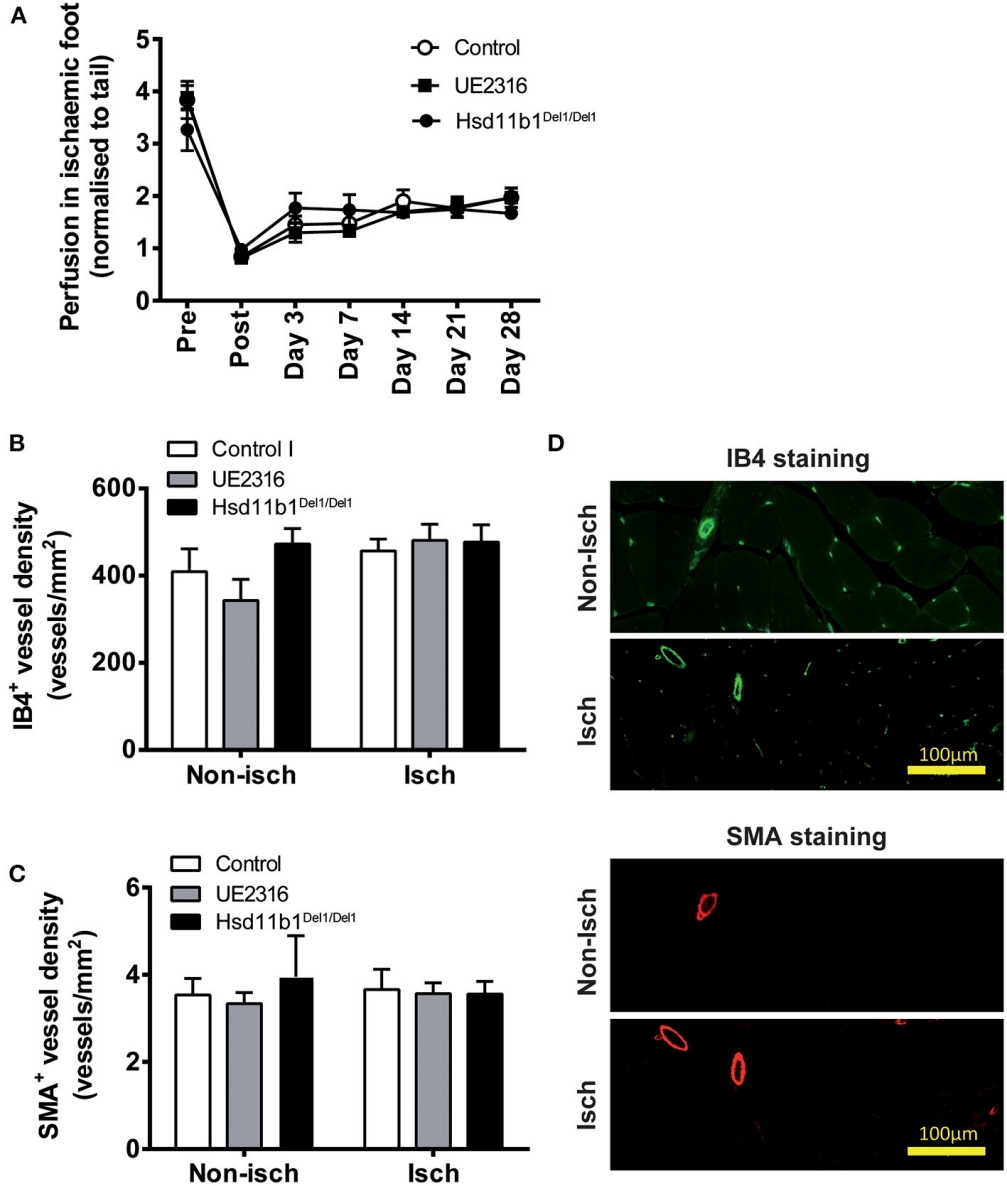
UE2316 treatment and 11βHSD1 deficiency had no effect on hindlimb ischaemia in aged mice. **(A)** Unilateral femoral artery ligation produced a dramatic reduction in hindlimb perfusion with a blunted reperfusion over 28 days. Neither UE2316 treatment nor 11βHSD1 deficiency affected post-ischaemic reperfusion **(A)**, capillary density **(B)** or artery density **(C)** in gastrocnemius muscles. **(D)** Representative images of capillaries [Isolectin B4 (IB4); green] and arteries [α-smooth muscle actin (SMA); red] in gastrocnemius muscles. Symbols are mean ± SEM; columns are mean + SEM (*n* = 8–9).

Femoral artery ligation in these mice induced a rapid reduction in blood perfusion (as seen in younger mice) but reperfusion appeared blunted (Figure 4A) compared with that in the younger animals; remaining at ~50% of pre-occlusion blood flow at day 28 (compared with ~70–80% in young mice). Neither UE2316 treatment nor 11βHSD1 deficiency had any effect on post-ischaemic reperfusion (Figure 4A). Furthermore, neither UE2316 treatment nor 11βHSD1 deficiency altered vascular density (Figures 4B–D) or transcriptomic expression profile (Supplementary Figure 3C) in ischaemic gastrocnemius muscle in aged mice 28 days following femoral artery ligation.

### Growth of Collateral Arteries Correlated With Rapid Reperfusion in Ischaemic Hindlimb

11βHSD1 deletion or inhibition had a clear proangiogenic effect in *ex vivo* angiogenesis and *in vivo* inflammatory angiogenesis. However, they had no effect on either vascular density or reperfusion in the mouse model of hindlimb ischaemia. Therefore, it was important to investigate the contribution of other vascular remodeling mechanisms (e.g., arteriogenesis) to post-ischaemic reperfusion. 3-Dimensional imaging of the hind limb (Supplementary Figures 4A–G) demonstrated that reperfusion of the hind limb in the 1st week following surgery was associated with the growth of collateral arteries in the upper thigh. Collateral arteries were first identifiable at Day 3. The maximum diameter of collateral arteries was nearly doubled at day 7 vs. day 3 (141 ± 2 μm vs. 69 ± 4 μm; *p* < 0.01 by Student's *t*-test). Prior to surgery, the femoral artery was the main artery supplying blood to the hind limb (Supplementary Figure 4H). Therefore, following ligation, the rapid establishment of a collateral circulation (through pre-existing vessels) makes a significant contribution to reperfusion of the hind limb. Typically, pre-existing small branches of the popliteal artery quickly grew into big, often tortuous, collateral arteries and connected to the vascular network in the upper thigh.

## Discussion

Impaired angiogenesis in patients with CLI has been suggested as an important mechanism contributing to poor reperfusion (2–4). However, the clinical benefit of VEGF-based therapeutic angiogenesis is inconclusive (2, 6, 7, 35). In this study, we investigated an alternative strategy to promote angiogenesis by blocking local reactivation of glucocorticoids. Whilst inhibition or deletion of 11βHSD1 increased *ex vivo* angiogenesis and *in vivo* inflammatory angiogenesis, this was not translated into an improvement in post-ischaemia reperfusion in the hindlimb. By contrast, the reperfusion in the 1st week was associated with rapid growth of collateral arteries in the ischaemic leg. These results suggest that enhanced angiogenesis by 11βHSD1 blockage is insufficient to improve reperfusion in a mouse model of hindlimb ischaemia.

Initially, this study demonstrated that inhibition 11βHSD1 activity is a valid strategy to stimulate angiogenesis both *ex vivo* and *in vivo*. The aortic ring assay examined the direct impact on angiogenesis (including endothelial sprouting and tube formation) in the absence of systemic influences. Consistent with a previous study (8), corticosterone inhibited angiogenesis both in wild type control and in Hsd11b1^Del1/Del1^ mice, whereas its inert metabolite 11DHC only inhibited angiogenesis in the presence of functional 11βHSD1, confirming that inhibition of angiogenesis by 11DHC is dependent on its conversion to corticosterone by 11βHSD1. Similar pro-angiogenic effects of inhibition or deletion of 11βHSD1 have been reported in adipose tissue and infarcted myocardium (18–21).

The sponge implant model was used to investigate inflammatory angiogenesis *in vivo* (8, 36). Notably, formation of new blood vessels in the sponge implants was associated with extensive leukocyte invasion (8) and prolonged expression of inflammatory genes at 21 days. The vascular density in sponge implants was significantly increased in Hsd11b1^Del1/Del1^ mice vs. wild type control. Zhang et al. reported that 11β-HSD1 in macrophages plays a dominant role in regulation of inflammatory angiogenesis in the sponge implant (36). This is consistent with the pro-inflammatory and proangiogenic effects of 11βHSD1 deficiency in a murine model of myocardial infarction which is characterized by prominent macrophage accumulation in the infarct border zone (8, 20). 11βHSD1 deficiency abolished glucocorticoid reactivation in the heart, leading to increased inflammatory cell infiltration, production of the pro-angiogenic chemokine IL-8, and subsequent endothelial cell proliferation (20). As such the anti-inflammatory effect of glucocorticoids may serve as an important mechanism to supress pathological angiogenesis (20, 36, 37).

By contrast, pharmacological inhibition of 11βHSD1 did not promote angiogenesis in the sponge implants. This has similarities with a recent investigation from our own group which demonstrated that administration of UE2316 did not increase angiogenesis in murine models of squamous cell carcinoma and pancreatic ductal adenocarcinoma (unpublished data). UE2316 is a new generation of 11βHSD1 antagonist with higher selectivity and potency. A single oral dose of UE2316 (10 mg/kg) can achieved an >60% inhibition of 11βHSD1 activity in brain tissue in 4 h whereas chronic treatment with UE2316 (10 mg/kg/day *via* osmotic minipump for 3–4 week) maintained a inhibition at 35–65% (29, 38). The UE2316 dosage in this study (0.0175% w/w blended in chow diet; with an estimation of 27 mg/kg/day) was adopted from a study on Alzheimer's disease which has demonstrated a therapeutic efficacy of EU2316 on cognitive function (29). However, it is quite possible that UE2316 (27 mg/kg/day) could not achieve complete inhibition of 11βHSD1 and the residual activity of 11βHSD1 is till sable to regenerate enough glucocorticoids to suppress angiogenesis. Further investigation is required to determine whether a higher dose of UE2316 is required to promote *in vivo* angiogenesis.

Strikingly, the pro-angiogenic effects obtained by blocking 11βHSD1 activity did not lead to improvements in reperfusion in the murine model of hindlimb ischaemia. In this model, femoral artery ligation induces acute ischaemia in the hindlimb (the calf and foot pad in particular). This stimulates extensive vascular remodeling to reperfuse the ischaemic tissue, including arteriogenesis in the upper thigh and angiogenesis in the lower leg. Establishment of collateral circulation restores upstream blood supply whilst angiogenesis reduces local perfusion resistance (39). We and others have demonstrated that arteriogenesis is an early event in post-ischaemic vascular remodeling that mediates rapid reperfusion in the 1st week (40), whereas angiogenesis is initiated relatively late. In a rat hindlimb ischaemia model, an increase in capillary-to-muscle fiber ratio was only detected after 35 days (41–43). In the current study, post-ischaemic reperfusion peaked at 7–14 days in young adult mice, suggesting that arteriogenesis, rather than angiogenesis, was the dominant mechanism driving reperfusion in this model. The rapid reperfusion in young mice, therefore, may obscure the role of angiogenesis in hindlimb ischaemia.

In order to clarify whether blocking 11βHSD1 is a valid strategy to improve post-ischaemic reperfusion via angiogenesis, we repeated the hindlimb ischaemia study using aged mice, since aging is associated with impaired arteriogenesis and angiogenesis and CLI is a condition associated with older age-groups (3, 4, 44, 45). Arteriogenesis in aged mice is severely impaired following hindlimb ischaemia, leaving angiogenesis to play a more important role in reperfusion (45). In addition, angiogenesis is also severely compromised in mice aged 20–40 weeks vs. mice aged 4–12 weeks (44). As such, we hypothesized that pro-angiogenic therapy would have a more prominent impact on post-ischaemic reperfusion in aged vs. young mice. In a cohort of mice aged 33 weeks, we found that arteriogenesis-mediated reperfusion in the 1st week was clearly blunted. However, blockage of 11βHSD1 still had no effect on vascular density or reperfusion at 28 days. These data are inconsistent with the demonstration that 11βHSD1 deficiency promoted angiogenesis in the sponge implant and in acute myocardial infarction (20). A notable difference between hindlimb ischaemia and the other two models is the scale of inflammation which is a strong stimulus of pathological angiogenesis. The low inflammatory status of the hindlimb ischaemia model is well-recognized. A published protocol of the femoral artery ligation model suggests to carefully avoid para-surgical bleeding as the clots will induce unnecessary inflammation and interfere the outcome of ischaemia-induced angiogenesis (39). In the current study, we haven't found any obvious muscle fiber damage or inflammatory cell invasion in the gastrocnemius muscle in either young or aged mice. The mRNA expression of inflammatory makers was barely detectable in ischaemic hindlimb, but remained elevated in sponge implants. Similarly to animal studies, clinical biopsy also demonstrated that gastrocnemius muscles from CLI patients were viable and histologically intact, comparable to samples from non-ischaemic legs (46, 47).

By contrast, cardiac muscle is extremely vulnerable to ischaemia as the oxygen consumption in normal myocardium is ~100 times higher than that in resting skeletal muscle (48, 49). Coronary ligation model induces rapid tissue death in the affected myocardium, which in turn triggers prominent inflammation, macrophage recruitment and angiogenesis in the infarct border zone. As such, it is hard to expect that the angiogenesis in ischaemic leg could reach a level comparable to infarcted myocardium. Further investigation is required to understand the interaction between ischaemia-induced inflammation, tissue damage, glucocorticoid generation and vascular remodeling.

Taken together, the data generated in this investigation demonstrate that glucocorticoids reduce *ex vivo* angiogenesis via direct inhibition of endothelial cell sprouting and tube formation, and suppress *in vivo* angiogenesis in a sponge implant model. However, 11βHSD1-deletion or inhibition do not increase vascular density or reperfusion in a murine model of hindlimb ischaemia which exhibited negligible chronic inflammation. This study provides a new line of evidence, in addition to the VEGF-based therapeutic angiogenesis (2, 6, 7), questioning the effectiveness of promoting angiogenesis alone as a therapeutic strategy for use in CLI. Future investigations on post-ischaemic reperfusion should consider the role of other aspects of systemic vascular remodeling, especially arteriogenesis and collateral formation.

## Data Availability Statement

The original contributions presented in the study are included in the article/Supplementary Material, further inquiries can be directed to the corresponding author/s.

## Ethics Statement

The animal study was reviewed and approved by the University of Edinburgh Ethical Review Committee (Animal Welfare and Ethical Review Body).

## Author Contributions

JW, EM, CD, BW, and PH designed the investigation. All authors contributed to design, analysis and interpretation of the experimental work which was performed by JW, EM, CD, and PH. JW drafted the manuscript which was reviewed and edited by all authors. All authors contributed to the article and approved the submitted version.

## Funding

This work was supported by the British Heart Foundation (Project Grant: PG/15/10/31277; and Studentship to CD).

## Conflict of Interest

The authors declare that the research was conducted in the absence of any commercial or financial relationships that could be construed as a potential conflict of interest.

## Publisher's Note

All claims expressed in this article are solely those of the authors and do not necessarily represent those of their affiliated organizations, or those of the publisher, the editors and the reviewers. Any product that may be evaluated in this article, or claim that may be made by its manufacturer, is not guaranteed or endorsed by the publisher.

## Supplementary Material

Supplementary Figure 1 Schematic figure for experiment design.

Supplementary Figure 2 11βHSD1 deficiency or UE2316 treatment did not alter food intake, body weight or internal organ weights of experimental animals. In both young **(A)** and aged **(B)** mice, the daily consumption of the UE2316-blended chow diet was the same as the control diet. The dose of UE2316 was estimated from averaged daily food intake. The body weight and internal organ weight were not affected by either genetic ablation of 11βHSD1 or UE2316 treatment.

Supplementary Figure 3 11βHSD1 deficiency and UE2316 treatment had limited impact on angiogenesis-related gene expression profile in hindlimb ischaemia and sponge implant models. **(A)** In the sponge implant model, 11βHSD1 deficiency or UE2316 (27 mg/kg/day) treatment did not affect the gene expression profile in sponge implants. The expression of pro-inflammatory genes (*Ccl2, Il1b, Il6, and Tnf)* in sponge implants remained detectable at post-graft day 21. **(B,C)** In the hindlimb ischaemia model, the expression of pro-inflammatory genes (*Ccl2, Il1b, Il6, and Tnf)* in gastrocnemius muscle were barely detectable at post ischaemic day 28. 11βHSD1 deficiency had no impact on the gene expression profile, whereas statistical differences were detected in the expression levels of *Vegfa* and *Cnn1* following UE2316 treatment. Bars represent mean + SEM. *P* < 0.05, by two-way ANOVA plus Bonferroni's *post-hoc* test (*n* = 9–10 for panel **A**, *n* = 6–9 for panel **B**, *n* = 10 for panel **C**).

Supplementary Figure 4 Rapid reperfusion in ischaemic hind limb is accompanied by growth of collateral arteries. The vasculature in the mouse hindlimb was visualized by combining resin casting and Optical Projection Tomography (OPT) imaging **(A–E)**, whilst blood flow in the foot was quantified using Laser Doppler **(F)** at 5 time points: pre-surgery (Pre), immediately post-surgery (Post), and then at day 1, day 3, and day 7 post surgery. Representative heatmaps of foot pad perfusion are inserted in the bottom-right corner of **(A–E)**. ^*^*P* < 0.05 vs. Post, by one-way ANOVA plus Bonferroni's *post-hoc* test. **(G)** The diameters of collateral arteries were measured using ImageJ. The maximum diameter of collateral arteries was 69 ± 4 μm at day 3 and 141 ± 2 μm at day 7. ^**^*P* < 0.01, by unpaired Student's *t*-test (*n* = 3). **(H)** The perfusion pattern and typical collateral growth was summarized in a schematic figure.
