# Supplementary figures and images for: Enhanced Angiogenesis by 11βHSD1 Blockage Is Insufficient to Improve Reperfusion Following Hindlimb Ischaemia
Source: Front Cardiovasc Med. 2022 Jan 12;8:795823. doi: 10.3389/fcvm.2021.795823 (PMC8790072; doi:10.3389/fcvm.2021.795823)

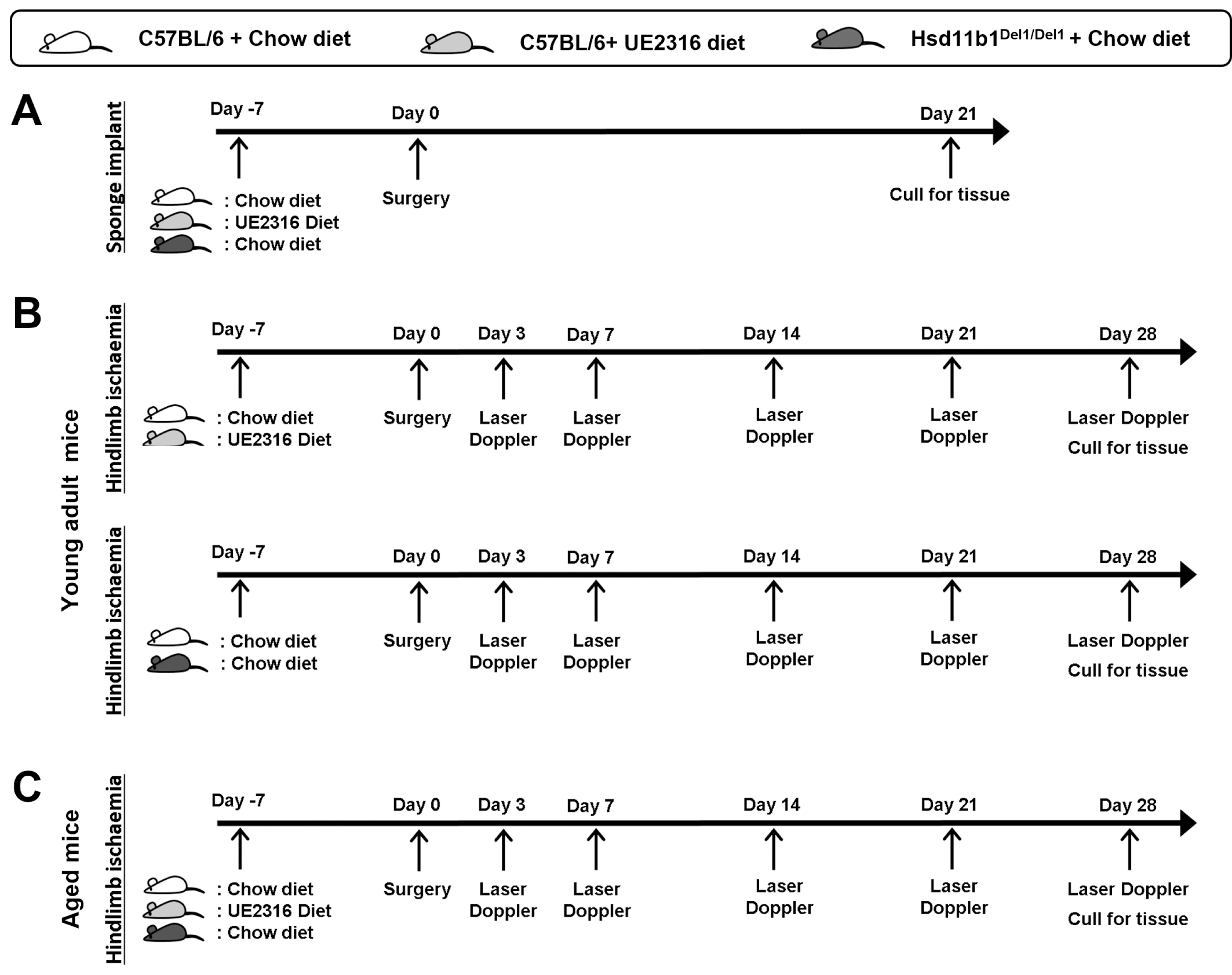

Supplement: Supplementary Figure 1 — Schematic figure for experiment design. [file Image_1.TIF]

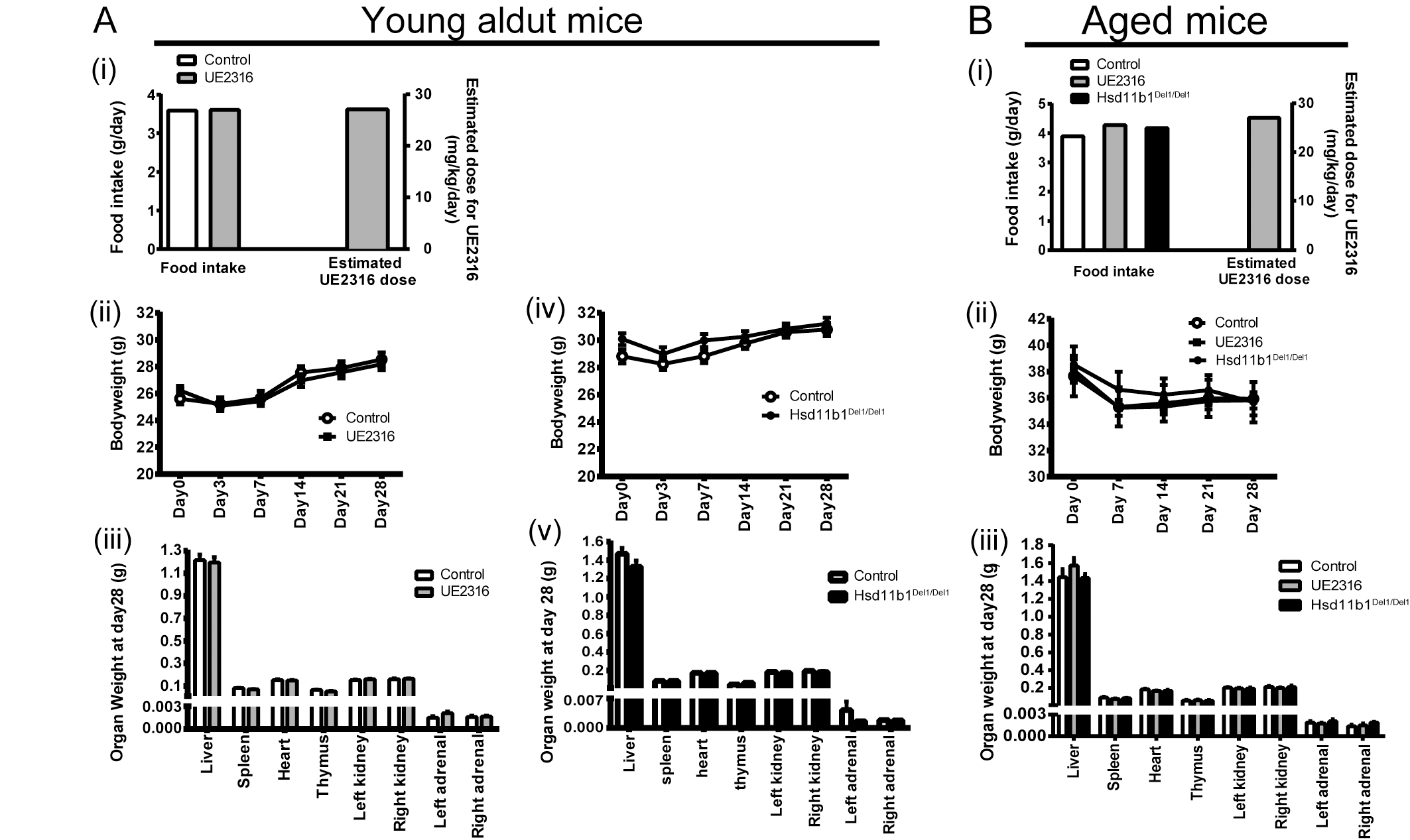

Supplement: Supplementary Figure 2 — 11βHSD1 deficiency or UE2316 treatment did not alter food intake, body weight or internal organ weights of experimental animals. In both young (A) and aged (B) mice, the daily consumption of the UE2316-blended chow diet was the same as the control diet. The dose of UE2316 was estimated from averaged daily food intake. The body weight and internal organ weight were not affected by either genetic ablation of 11βHSD1 or UE2316 treatment. [file Image_2.TIF]

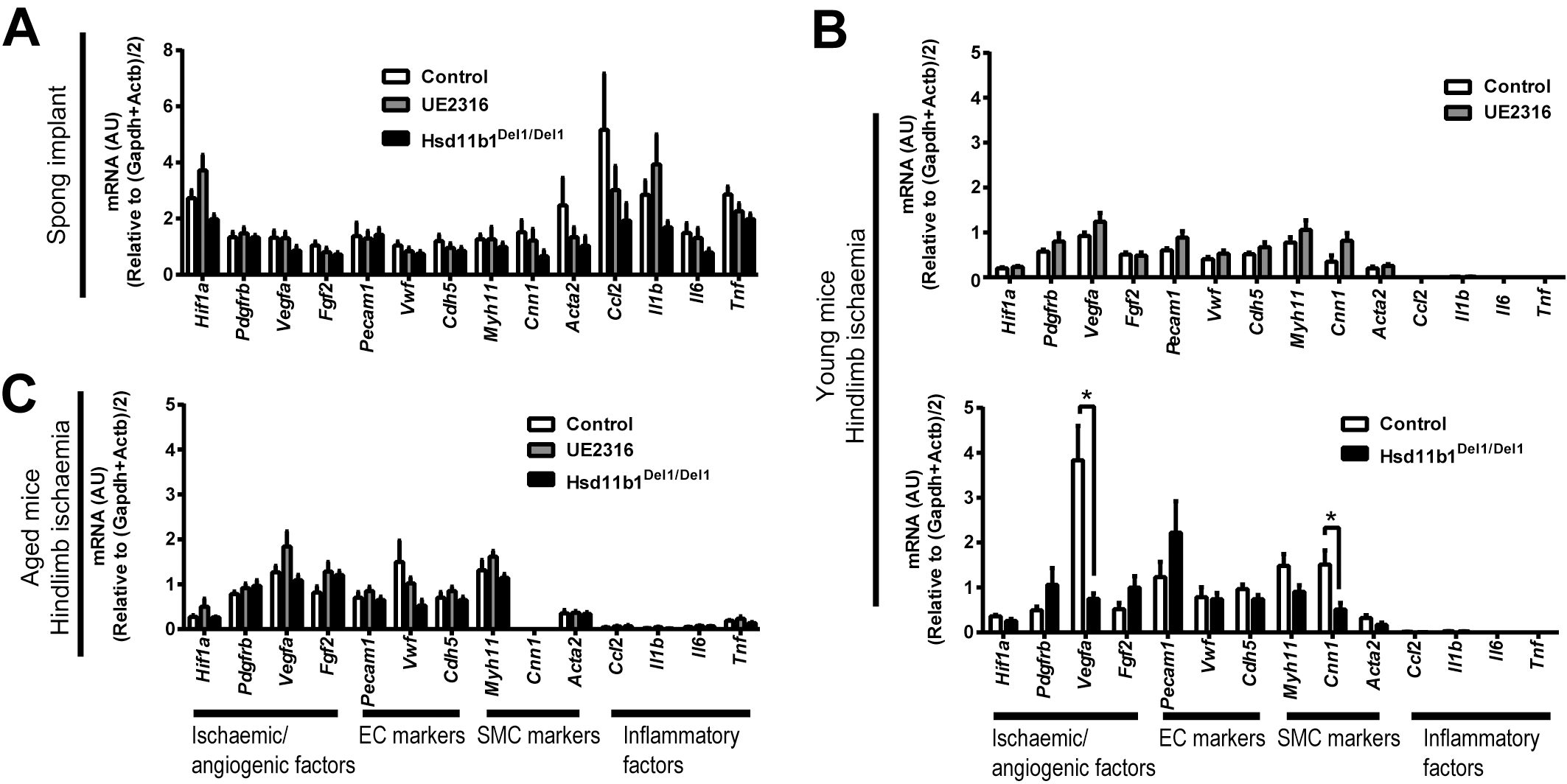

Supplement: Supplementary Figure 3 — 11βHSD1 deficiency and UE2316 treatment had limited impact on angiogenesis-related gene expression profile in hindlimb ischaemia and sponge implant models. (A) In the sponge implant model, 11βHSD1 deficiency or UE2316 (27 mg/kg/day) treatment did not affect the gene expression profile in sponge implants. The expression of pro-inflammatory genes (Ccl2, Il1b, Il6, and Tnf) in sponge implants remained detectable at post-graft day 21. (B,C) In the hindlimb ischaemia model, the expression of pro-inflammatory genes (Ccl2, Il1b, Il6, and Tnf) in gastrocnemius muscle were barely detectable at post ischaemic day 28. 11βHSD1 deficiency had no impact on the gene expression profile, whereas statistical differences were detected in the expression levels of Vegfa and Cnn1 following UE2316 treatment. Bars represent mean + SEM. P < 0.05, by two-way ANOVA plus Bonferroni's post-hoc test (n = 9–10 for panel A, n = 6–9 for panel B, n = 10 for panel C). [file Image_3.TIF]

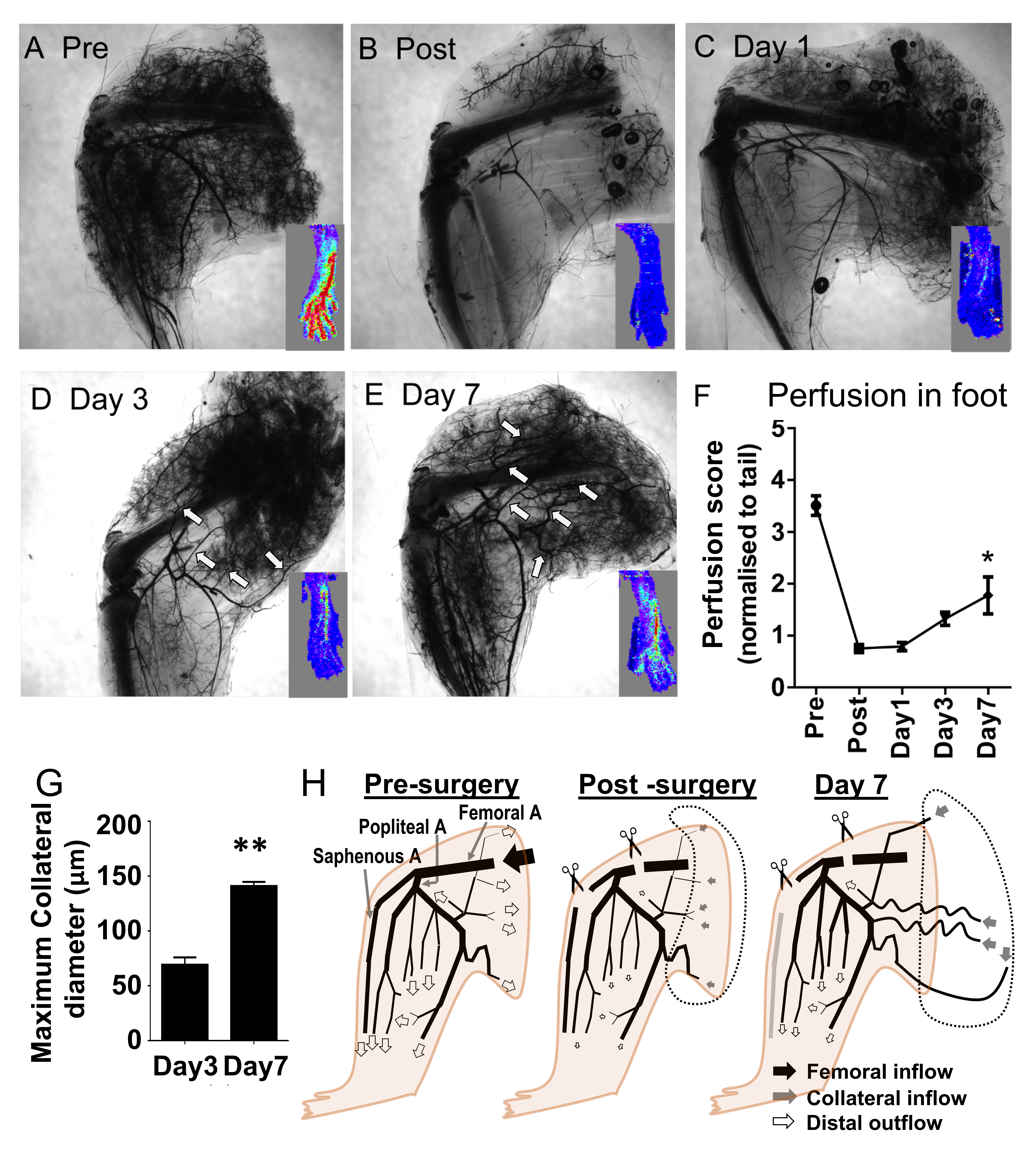

Supplement: Supplementary Figure 4 — Rapid reperfusion in ischaemic hind limb is accompanied by growth of collateral arteries. The vasculature in the mouse hindlimb was visualized by combining resin casting and Optical Projection Tomography (OPT) imaging (A–E), whilst blood flow in the foot was quantified using Laser Doppler (F) at 5 time points: pre-surgery (Pre), immediately post-surgery (Post), and then at day 1, day 3, and day 7 post surgery. Representative heatmaps of foot pad perfusion are inserted in the bottom-right corner of (A–E). *P < 0.05 vs. Post, by one-way ANOVA plus Bonferroni's post-hoc test. (G) The diameters of collateral arteries were measured using ImageJ. The maximum diameter of collateral arteries was 69 ± 4 μm at day 3 and 141 ± 2 μm at day 7. **P < 0.01, by unpaired Student's t-test (n = 3). (H) The perfusion pattern and typical collateral growth was summarized in a schematic figure. [file Image_4.TIF]
